# Pleiotropic effects of a *rel *mutation on stress survival of *Rhizobium etli *CNPAF512

**DOI:** 10.1186/1471-2180-8-219

**Published:** 2008-12-10

**Authors:** Kristien Braeken, Maarten Fauvart, Maarten Vercruysse, Serge Beullens, Ivo Lambrichts, Jan Michiels

**Affiliations:** 1 Centre of Microbial and Plant Genetics, Katholieke Universiteit Leuven, B-3001 Heverlee, Belgium; 2 Biomedical Research Institute, Hasselt University, B-3590 Diepenbeek, Belgium

## Abstract

**Background:**

The *rel *gene of *Rhizobium etli *(*rel*_*Ret*_), the nodulating endosymbiont of the common bean plant, determines the cellular level of the alarmone (p)ppGpp and was previously shown to affect free-living growth and symbiosis. Here, we demonstrate its role in cellular adaptation and survival in response to various stresses.

**Results:**

Growth of the *R. etli rel*_*Ret *_mutant was strongly reduced or abolished in the presence of elevated NaCl levels or at 37°C, compared to the wild type. In addition, depending on the cell density, decreased survival of exponentially growing or stationary phase *rel*_*Ret *_mutant cells was obtained after H_2_O_2_, heat or NaCl shock compared to the wild-type strain. Survival of unstressed stationary phase cultures was differentially affected depending on the growth medium used. Colony forming units (CFU) of *rel*_*Ret *_mutant cultures continuously decreased in minimal medium supplemented with succinate, whereas wild-type cultures stabilised at higher CFU levels. Microscopic examination of stationary phase cells indicated that the *rel*_*Ret *_mutant was unable to reach the typical coccoid morphology of the wild type in stationary phase cultures. Assessment of stress resistance of re-isolated bacteroids showed increased sensitivity of the *rel*_*Ret *_mutant to H_2_O_2 _and a slightly increased resistance to elevated temperature (45°C) or NaCl shock, compared to wild-type bacteroids.

**Conclusion:**

The *rel*_*Ret *_gene is an important factor in regulating rhizobial physiology, during free-living growth as well as in symbiotic conditions. Additionally, differential responses to several stresses applied to bacteroids and free-living exponential or stationary phase cells point to essential physiological differences between the different states.

## Background

*Rhizobium etli *is a Gram-negative soil bacterium that elicits nitrogen-fixing nodules on its leguminous host plant *Phaseolus vulgaris*, the common bean plant. Although the precise nutritional conditions under which the bacteroids thrive inside the nodule cells are still not known, the physiological state of the bacteroids needs to adapt to the prevailing conditions such as a microoxic and low pH environment, specific carbon and nitrogenous compounds and the presence of oxidative stress (reviewed by e.g. [1,2]).

RelA/SpoT homologues are involved in the regulation of the (p)ppGpp level in the cell. Research in *Escherichia coli *revealed that this molecule is important in the reorganization of the cellular metabolism during nutrient starvation [3-6]. Moreover, the importance of RelA/SpoT homologous proteins and the effector (p)ppGpp during starvation for nutrients or survival in the presence of specific exogenous stresses was demonstrated for a growing number of micro-organisms [5,7]. In recent years, it has become clear that (p)ppGpp is also required in complex physiological processes such as biofilm formation by *Listeria monocytogenes*, *E. coli *and *Streptococcus mutans *and developmental processes such as multicellular fruiting body formation by *Myxococcus xanthus *or sporulation by *Bacillus subtilis*. Also, RelA/SpoT homologous proteins have been reported to be important for the interaction of bacteria, either pathogenic or beneficial, with their eukaryotic host [7]. In symbiotic bacteria, a RelA/SpoT homologue was first characterized in *Sinorhizobium meliloti *[8,9]. An *S. meliloti rel*_*Sme *_mutant is unable to induce the stringent response and overproduces succinoglycan, an exopolysaccharide that is important for infection of its host plant *Medicago sativa*. Moreover, *rel*_*Sme *_is required for nodule formation on its host [8]. It was subsequently demonstrated in *R. etli *that the inactivation of *rel*_*Ret *_strongly affects symbiosis with its host *Phaseolus vulgaris *[10,11]. Plants nodulated by a *R. etli rel*_*Ret *_mutant have a strongly reduced nitrogen fixation activity. Moreover bacteroid morphology is altered in the *rel*_*Ret *_mutant strain. These findings indicate that adjustment of rhizobial physiology may be a key process to establish a successful symbiosis.

Although *relA/spoT *genes have been described in a large number of bacteria, extensive phenotyping of the corresponding mutants has presently been carried out in only a limited number of species. Because of its putative involvement in stress survival, we performed a detailed examination of the *R. etli rel*_*Ret *_mutant in response to salt, temperature, oxidative and stationary phase stresses. In addition, as the physiological status of rhizobial bacteroids is currently not fully understood, phenotyping of wild-type and *rel*_*Ret *_mutant bacteroids was performed. Our results indicate a prominent role for (p)ppGpp in *R. etli *survival in the presence of specific stress conditions and in the adaptation of the bacterium to the endosymbiotic bacteroid state.

## Results

### Growth in the presence of chronic stress

In a previous study, we demonstrated a clear impact of a *rel*_*Ret *_mutation on growth in complex as well as in defined minimal medium [10]. To further explore the role of *rel*_*Ret *_in the presence of various stresses, specific growth experiments were performed.

**Figure 1:**
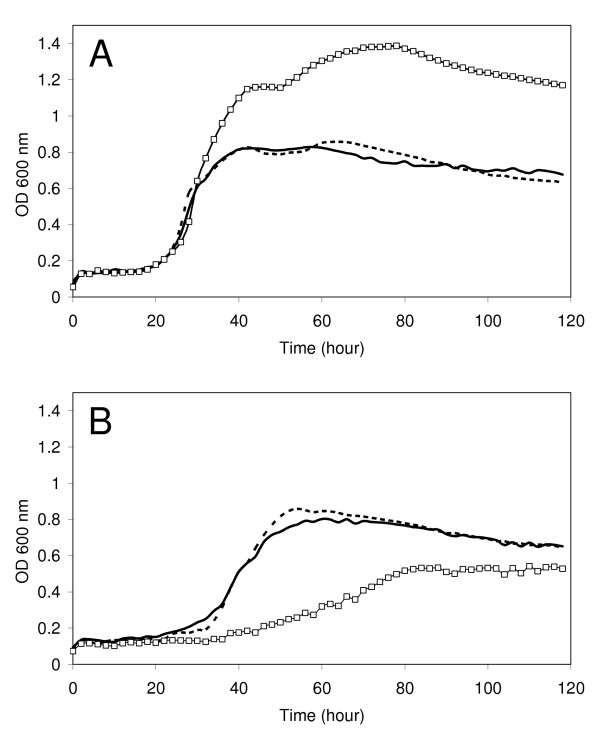
**Growth in TY medium supplemented with increasing concentrations of NaCl.** Growth curves of wild-type *R. etli *CNPAF512 (black), *rel*_*Ret *_mutant CMPG8705 (squares) or CMPG8705 complemented with pCMPG8715 (black dashed line) were measured in a Bioscreen C for standard TY medium (A) and TY medium supplemented with 0.075 M NaCl (B). Standard deviations are typically 0.03–0.04 (5 repeats). The experiment was repeated 3 times and a representative graph is shown.

To assess the effect of NaCl on *R. etli *growth, increasing amounts of NaCl were added to standard TY medium. Almost no effect was observed upon addition of concentrations below 0.05 M NaCl (data not shown). In the presence of 0.075 M NaCl, the lag phase of the wild-type strain increased slightly, whereas the *rel*_*Ret *_mutant CMPG8705 displayed a strongly extended lag phase and a considerable increase in generation time (Fig. 1). Moreover, in contrast to standard TY medium, optical densities of CMPG8705 cultures in stationary phase were lower compared to the wild type. The addition of 0.1 M NaCl completely inhibited growth of the *rel*_*Ret *_mutant CMPG8705, while the wild type displayed increased lag phase and generation time (data not shown). These phenotypes were almost completely complemented upon introduction of pCMPG8715. Above 0.15 M NaCl, none of the strains could grow (data not shown). Similar effects were observed when cells were grown in AMS minimal medium containing 10 mM succinate as carbon source (data not shown).

**Figure 2:**
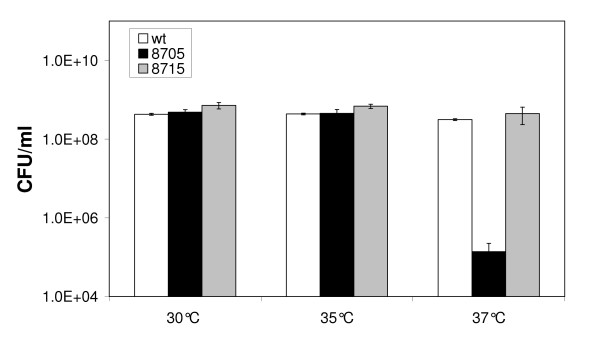
**Temperature sensitivity of the *rel*_*Ret *_mutant.** The wild type (white), *rel*_*Ret *_mutant CMPG8705 (black) and the complemented strain CMPG8705/pCMPG8715 (gray) were grown to an OD_600 _of 0.3 and plated on TY agar plates incubated at temperatures of 30°C, 35°C and 37°C. Growth is expressed in CFU ml^-1^. The data are the means of three replicates. Bars represent standard deviations. Experiments were repeated three times and a representative graph is shown.

As thermosensitive growth of *relA/spoT *mutants has been reported in other organisms such as *E. coli *[12] and *Mycobacterium tuberculosis *[13], we determined the effect of temperature on growth of the *rel*_*Ret *_mutant. *R. etli *wild type, *rel*_*Ret *_mutant CMPG8705 and the complemented strain were grown to exponential (OD_600 _of 0.3), early stationary (24 h culture; data not shown) and late stationary phase (48 h culture; data not shown) in TY broth. From these cultures, dilution series were plated on TY agar plates, incubated for three days at various temperatures and scored for colony forming units (CFUs). No differences were observed at 25°C, 34°C (data not shown) and 35°C compared to growth at 30°C (Fig. 2). Also, incubation for 24 h at 4°C followed by three days of incubation at 30°C did not affect numbers of CFUs. However, at 37°C, the *rel*_*Ret *_mutant formed significantly less colonies. The plating efficiency (plate count at 37°C divided by plate counts at 30°C) was 75% or higher for the wild type and complemented strain in the three conditions tested (exponential, early and late stationary phase). In the mutant strain, the plating efficiency for exponential and early stationary phase cultures was 0.02–0.03%, and declined to 0.002% at the 48 h time point (Fig. 2; data not shown). However, at the latter time point, the number of CFUs also declined in the mutant strain grown at 30°C, possibly contributing to the increased reduction. Increased temperature sensitivity of the *rel*_*Ret *_mutant was also confirmed in AMS succinate medium (data not shown).

### Survival in the presence of acute stress

In addition to chronic stress growth experiments, survival of wild-type *R. etli*, *rel*_*Ret *_mutant CMPG8705 and CMPG8705 complemented with pCMPG8715 was determined in the presence of oxidative stress, salt stress, heat shock and cold for cells harvested throughout the growth curve, varying from early logarithmic to late logarithmic-early stationary phase. Cells at different optical densities were challenged with one of the various stresses for a defined time period and the number of surviving CFUs was determined after 3 days of incubation at 30°C.

**Figure 3:**
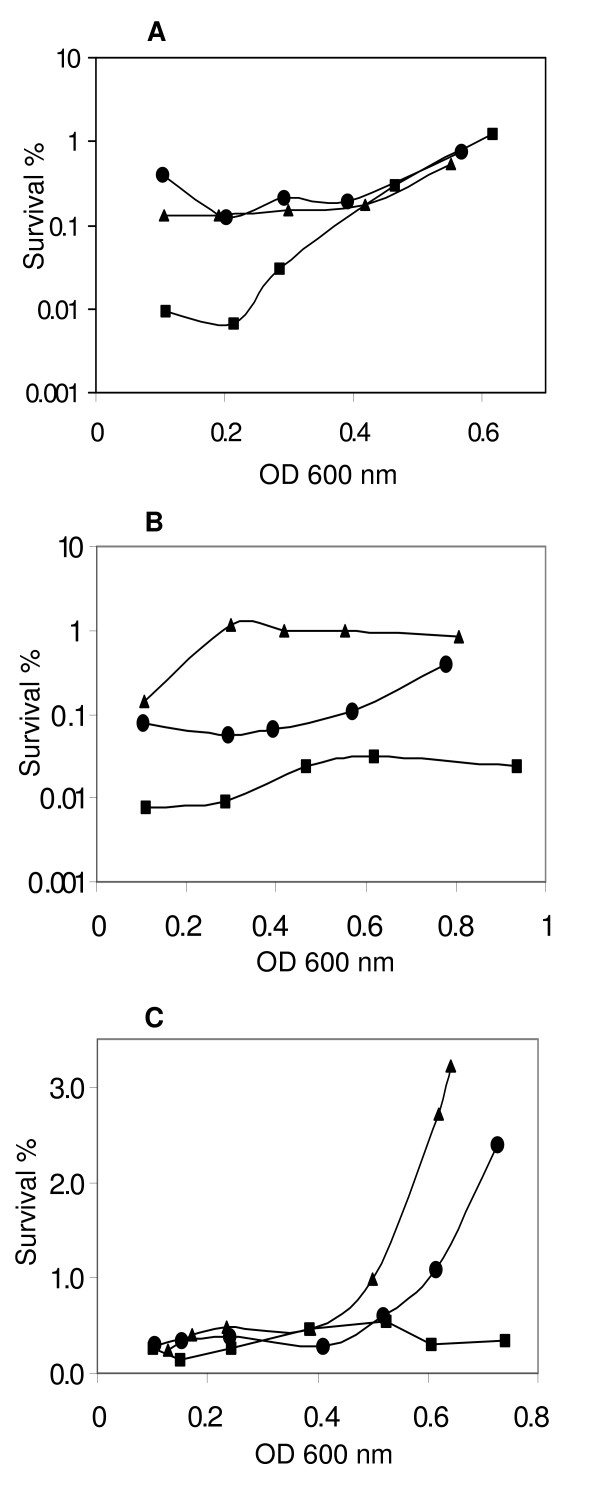
**Oxidative, heat and salt stress sensitivities at different cell densities.** Strains used are wild-type *R. etli *CNPAF512 (circles); *rel*_*Ret *_mutant CMPG8705 (squares) and the complemented strain CMPG8705/pCMPG8715 (triangles) grown in TY medium. Results are presented as percentages, calculated as the number of CFUs in the stressed sample relative to the number of CFUs of the corresponding control sample. Control samples were incubated at 30°C for the same time period as treated samples. (A) Samples were incubated for 30 min in the presence of 10 mM H_2_O_2 _in a 30°C water bath. The experiment was repeated 3 times and a representative graph is shown. (B) Samples were incubated for 60 min in a 45°C water bath. The experiment was repeated 2 times and a representative graph is shown. (C) Samples were incubated for 6 hours in a 30°C shaking incubator in the presence of a final concentration of 2.5 M NaCl.

To assess survival under oxidative conditions, cells were incubated for 30 min (30°C) in the presence of 10 mM H_2_O_2 _(Fig. 3A). Unsurprisingly, resistance of all strains is most elevated at the late exponential – early stationary phase, reaching survival percentages of 8–15% for OD_600 _above 0.65 (data not shown). No significant differences can be observed between the strains under study at this stage. However, at OD_600 _values below 0.3, survival of the *rel*_*Ret *_mutant strain was consistently about 10-fold lower compared to the wild type and the complemented strain (significant at p < 0.05).

The effect of heat stress was first determined by shifting the cells to 45°C for 30 min. Wild-type and *rel*_*Ret *_mutant strains showed 2–10% survival depending on the cell density, but did not differ significantly. By contrast, the complemented strain showed survival levels of 50–100% (data not shown). After 60 min incubation, the survival percentages of the *rel*_*Ret *_mutant were 3- to 10-fold lower than those of the wild type (Fig. 3B). The complemented strain displayed increased survival reaching about 1% at OD_600 _above 0.3. In addition, the effect of prolonged incubation at low temperature was tested. Samples corresponding to a range of OD_600 _values were incubated without shaking at 4°C. Viability remained essentially unchanged during the first 20 days. At 27 days of incubation, viability dropped (about 10-fold), but no differences were observed between the strains (data not shown).

Finally, given the effects of NaCl on growth, survival after exposure to high concentrations of NaCl was examined. NaCl was added to the cell suspensions to a final concentration of 2.5 M. After 6 h incubation at 30°C, a clear impact on survival was observed (Fig. 3C). The *rel*_*Ret*_ mutant showed similar or slightly lower survival compared to the wild type during different repeats of the experiment. Only during late exponential phase, differences are more pronounced (significant at p < 0.05).

### Survival of reisolated bacteroids during acute stress

To gain more insight into effects mediated by *rel*_*Ret *_in the bacteroids, stress resistance of isolated bacteroids was investigated at 3, 5 and 6 weeks after inoculation. The data in Table 1 indicate that bacteroids are still able to redifferentiate into free-living, growing bacteria. However, in contrast to the wild type, the number of *rel*_*Ret *_CFUs per gram nodule weight was approximately 10-fold lower for all time points analyzed.

**Table 1:** Survival of re-isolated bacteroids following stress application

**Conditions tested**	**Strains**		
	***Wild type***	***CMPG8705***	***CMPG8705/*** ***pCMPG8715***

**10 mM H_2_O_2 _(30 min)^a^**	97.22% (4.81%)	26.25% (5.30%)	95.00% (7.07%)

**10 mM H_2_O_2_(60 min)**	91.24% (9.11%)	20.00% (7.07%)	95.00% (7.07%)

**45°C (30 min)**	8.11% (0.58%)	19.92% (4.83%)	13.00% (4.24%)

**45°C (60 min)**	1.41% (0.29%)	9.00% (1.41%)	3.40% (1.98%)

**2.5 M NaCl (90 min)**	12.97% (4.52%)	26.00% (2.12%)	10.76% (1.47%)

**2.5 M NaCl (240 min)**	6.10% (1.88%)	15.67% (3.77%)	6.90% (3.82%)

^a ^As described in the materials and methods section, bacteroids isolated from three different plants 35 days after inoculation were pooled for one sample. Three samples were tested for each strain (9 plants). Stresses applied were 10 mM H_2_O_2 _for 30 or 60 min, heat stress (45°C) for 30 or 60 min and high salt concentration (final concentration 2.5 M NaCl) for 90 or 240 min. Standard deviations are in parentheses. Values (CFU per gram nodule) of the control sample are 2.40E+09 (8.95E+08) (wild type), 2.20E+08 (4.69E+07) (CMPG8705) and 1.05E+09 (2.26E+08) (CMPG8705/pCMPG8715). A representative data set of the results obtained during repeats of the experiment is shown.

Oxidative stress, resulting from the presence of H_2_O_2_, hydroxyl and superoxide radicals, is an important stress factor in functioning nodules as reactive oxygen species (ROS) levels are elevated and increase even further during nodule senescence [14,15]. Therefore, the resistance against oxidative stress was determined for bacteroids isolated at various time points. As can be seen from Table 1, the wild type and complemented strain exhibit close to 100% resistance to a 30 or 60 min treatment with 10 mM H_2_O_2 _when isolated from nodules harvested 5 or 6 (data not shown) weeks post inoculation. In contrast, the survival percentage of the *rel*_*Ret *_mutant was only 20–26% (significant at p < 0.05).

Additionally, heat and salt stress were applied to the bacteroids. Surprisingly, resistance to both stresses at 5 weeks post inoculation was consistently found to be slightly higher in the mutant strain (Table 1; significant at p < 0.05). This was also observed 3 weeks post inoculation (data not shown). However, as senescence proceeded, at 6 weeks post inoculation, differences in heat and salt resistance between the studied strains disappeared (data not shown).

### Long-term survival in complex and defined medium

**Figure 4:**
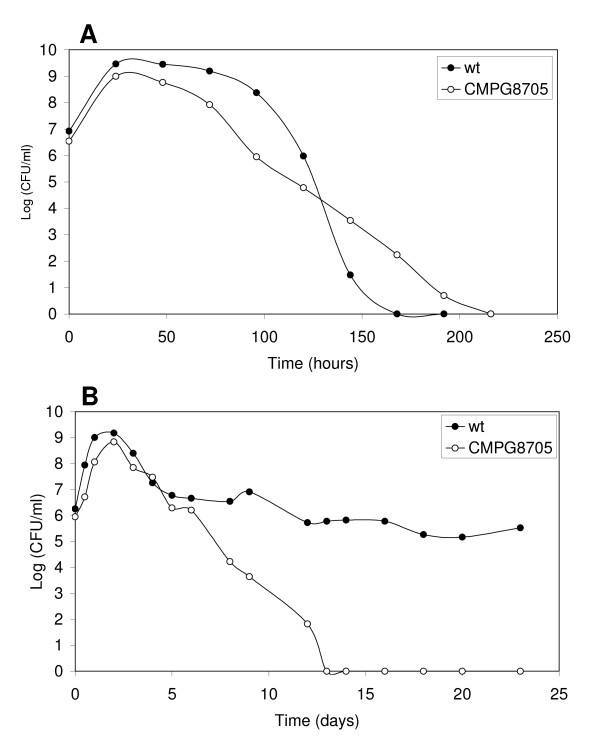
**Stationary phase survival.** Survival of wild-type *R. etli *CNPAF512 (•) and the *rel*_*Ret *_mutant CMPG8705 (*o*) 1/100 inoculated at OD 600 nm 0.4 in TY medium (A) and AMS medium containing 10 mM succinate (B). At regular time points, samples were taken and plated in duplicate. The logarithmic value of the number of CFU/ml is given. The graphs are representative of experiments carried out independently 2 or 3 times.

To study long-term survival, bacteria were grown in TY broth and analyzed at defined time points. After reaching stationary phase, CFUs of the *rel*_*Ret *_mutant initially decreased more rapidly compared to the wild type, leading to a difference of approximately 3 log units 3 days after entry into the stationary phase (Fig. 4A). However, after this time point, CFUs from the wild type rapidly dropped to zero. This is in contrast to the mutant displaying a more gradual decrease. Previous reports have described the phenomenon of substrate-accelerated death, i.e. cultures exhausted for a specific substrate yield a higher percentage of CFUs on medium without the traumatic substrate. Therefore, other media (AMS succinate, YEM medium), either in the presence or absence of antibiotics, and longer incubation times of the plates were tested for plate counts. However, none of these conditions allowed rescue of CFUs. Colonies of the *rel*_*Ret *_mutant at these late time points had retained their Sp-resistance and displayed growth curves indistinguishable from the mutant strain, indicating that they were derived from the mutant (data not shown).

In an attempt to determine if the effect on survival was associated with the medium used, the survival profile was also assessed in AMS medium containing 10 mM succinate (Fig. 4B). In contrast to what was observed using TY medium, the wild-type strain survived for a much longer time, reaching about 5 × 10^5 ^CFU ml^-1 ^23 days after inoculation. In contrast, the mutant strain displayed a rapid decrease of CFUs from about 5 days after inoculation. The limiting factor in nutrient deprivation in this medium is probably the carbon source, as the concentration of succinate was found to affect final optical densities reached in the stationary phase. In contrast, lowering the ammonium concentration from 10 mM to 2 mM did not affect the growth curve. These data suggest that the *rel*_*Ret *_mutant has stationary phase defects in this carbon-starved medium.

### Microscopic analysis of cells in stationary phase

**Figure 5:**
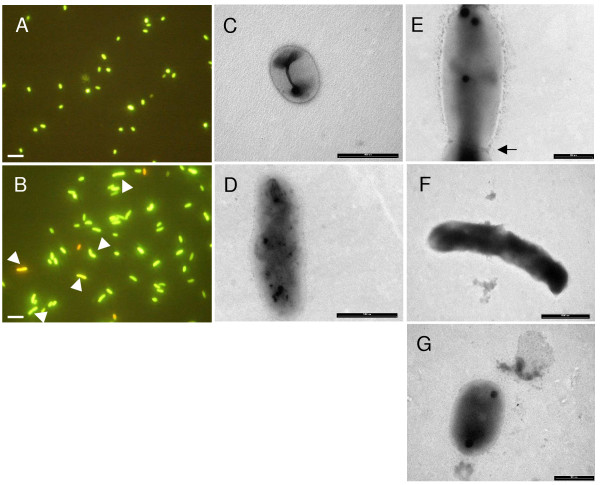
**Fluorescence and transmission electron microscopy observations of *R. etli *stationary phase cells and bacteroids.** Strains used are *R. etli *wild type (A, C and G) and the *rel*_*Ret *_mutant CMPG8708 (B, D, E, F). Panels A and B are fluorescence microscopic observation of stationary phase cells. Samples were coloured 120 h post inoculation (1/100 inoculated at OD 600 nm 0.4 in TY medium), using the LIVE/DEAD BacLight Bacterial Viability kit. Constrictions in several enlarged *rel*_*Ret *_mutant cells are indicated with a white arrow. Panels C, D and E are TEM observations of stationary phase bacteria at 5 days after inoculation in TY medium. The arrowhead indicates a constriction site, only observed for some cells in the mutant samples. Panels F and G are TEM observations of bacteroids isolated from the nodules 35 days after inoculation. Bar represents 5 μm (A, B), 1 μm (C, D, G), 0.5 μm (E, F).

To further unravel the observed survival phenotype during stationary phase in TY medium, samples were taken every 24 hours during the 48–144 h interval (Fig. 4) and observed via fluorescence light microscopy after vital staining. The enlarged cell morphology of the *rel*_*Ret *_strain compared to the wild type was obvious at all time points tested (Fig. 5A and 5B). Most of the cells (90%, based on the observation of approximately 500 cells) of both wild type and mutant stained with SYTO 9 at the 48 h time point, indicating that membrane integrity was not compromised. In contrast to the CFU values, this percentage remained approximately 90% for the wild type during the 48–144 h interval, and decreased only slightly (to 80%) in the mutant strain. However, in both strains, membranes finally started to disintegrate, as the number of propidium iodide staining cells rapidly increased upon further incubation reaching 60–70% after 2 weeks of incubation in TY medium.

Transmission electron microscopy analysis indicated that the wild type and *rel*_*Ret *_strain exhibited a similar morphology during the exponential phase (OD_600 _of 0.5; data not shown). At 120 h after inoculation, cells were still intact and the average length of the wild-type bacteria changed from 1.6 μm observed for the exponential phase to 1.2 μm (p < 0.01, student t-test). In contrast, the average length of the *rel*_*Ret *_mutant increased from 1.7 to 2.1 μm although the latter increase was not significant at p < 0.01 (Fig. 5C and 5D). Mutant cells also displayed a more dispersed electron density whereas this seemed much more concentrated at the poles in the wild-type cells. Furthermore, abnormally long cells showing a constriction in the middle were observed for the *rel*_*Ret *_mutant (Fig. 5B and 5E). Strikingly, also during symbiosis, a majority of the *rel*_*Ret *_mutant bacteroids isolated 5 weeks after inoculation were consistently larger but more electron dense compared to the wild type (Fig. 5F and 5G). These data are in agreement with previous results [10].

### Survival of stationary phase cultures during acute stress

**Figure 6:**
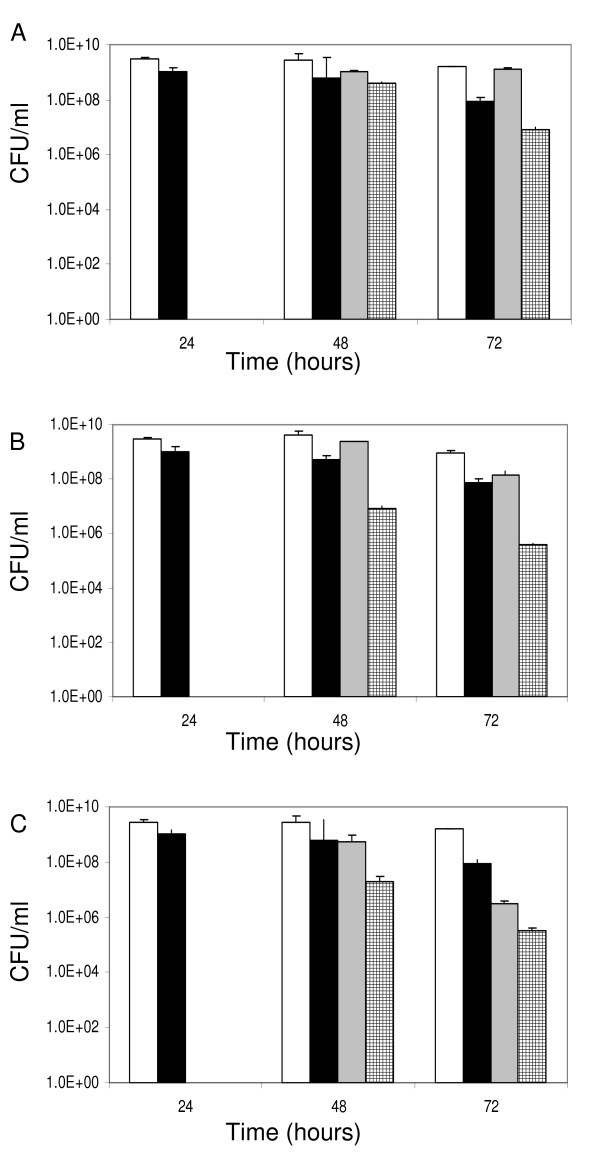
**Stress resistance of stationary phase TY cultures.** Stress resistance was analyzed at 48 and 72 h after inoculation. (A) Oxidative stress (10 mM H_2_O_2 _30 min), (B) NaCl 2.5 M 60 min, (C) Heat stress 45°C 30 min. Strains displayed are the wild-type untreated control (white), *rel*_*Ret *_untreated control (black), the wild-type survival (gray) and the *rel*_*Ret *_mutant survival (shaded) under the indicated stress condition. Samples were plated in triplicate and the experiment was repeated independently.

Finally, stress resistance of bacteria grown in TY broth for 48 or 72 h was assessed. As shown in previous tests, both strains were quite resistant against oxidative stress at the onset of the stationary phase and no differences were observed between the strains. In the late stationary phase (72 h), the decrease of resistance against oxidative stress was strongest in the mutant (Fig. 6). When salt stress was applied, the bacteria were generally more sensitive compared to during exponential growth. This effect was strongest in the *rel*_*Ret *_mutant. Finally, under temperature stress, the mutant strain displayed higher heat sensitivity compared to the wild type at the 48 h time point.

## Discussion

### Metabolic control and stress resistance

The alarmone (p)ppGpp likely plays a central role in adaptation of the *R. etli *metabolism as is reflected by the observed growth defects. Given the effect of different carbon sources, there is probably a link between the stringent response and the carbon status of the cell in *R. etli*. This is further supported by the survival of *R. etli *CNPAF512 in AMS minimal medium containing 10 mM succinate as the limiting nutrient. Production of (p)ppGpp controlled by carbon metabolism allows the cell to respond to conditions of carbon stress, and as a result would downregulate cellular metabolism. The molecular mechanism connecting the stringent response to the available carbon compounds in bacteria is still a major question to be resolved. Interestingly, differential expression of *rel*_*Ret *_in an *R. etli ptsA *mutant during symbiosis as well as under free-living aerobic growth was previously observed [10]. In *E. coli*, the link between the stringent response and the cellular carbon status is exerted through the SpoT protein. Recently, fatty acid metabolism has been shown to control the activity of SpoT [16].

In a growing number of bacteria, (p)ppGpp is associated with survival of stresses including general (stationary phase) stress as well as more specific ones [3,4,17]. In *R. etli*, mutation of *rel*_*Ret *_also affects heat and NaCl sensitivity, as well as resistance against oxidative stress depending on the growth phase. Moreover, sustained growth in the presence of high temperature and salt concentration was affected as well. Effects on osmotolerance have previously been reported for *L. monocytogenes *and *S. meliloti *[18,19], although the underlying basis is unclear. Here, salt tolerance of *R. etli *is specifically impaired at the late logarithmic-early stationary phase, so the aberrant cell morphologies observed might be more sensitive to the increased osmotic pressure in the medium. Temperature-sensitive growth has been described for *M. tuberculosis rel*_*Mtu *_[13], *V. cholerae *[20], and also coincides with decreased thermotolerance in *E. coli relA *mutants [12]. In the latter, this phenotype was osmoremedial. A possible explanation could be the involvement of heat shock proteins. However, overexpresion of σ^32 ^in *E. coli *did not relieve the observed phenotype. Induction of the heat shock response by (p)ppGpp has been a matter of debate in *E*. *coli*. Initially, Grossman *et al*. (1985) [21] reported that the stringent response induced heat shock gene expression. In contrast, Van Bogelen and Neidhardt (1990) [22] found that a *relA spoT *mutant displayed a modestly altered heat shock response and concluded that (p)ppGpp was neither sufficient nor absolutely necessary. The observation by Yang and Ishiguro (2003) [12] confirms that expression of heat shock proteins is not sufficient to relieve the temperature sensitivity exhibited by *relA *mutant strains. However, these authors identified *rpoB *mutants, previously shown to reverse amino acid auxotrophy, that suppressed the temperature phenotype. This indicates that the effect might be the consequence of a much more general aspect of (p)ppGpp-RNAP interaction. Besides *E. coli*, research in other bacteria, including *Streptococcus pyogenes *and *M. tuberculosis *points to (p)ppGpp-independent induction of heat shock genes [23,24]. To determine if more general aspects of the (p)ppGpp-mediated gene regulation are involved, it would be worthwhile testing if conserved *rpoB *suppressor mutations, as also identified in *S. meliloti *[9], affect salt sensitivity as well or whether introduction of these mutations in *R. etli *CNPAF512 reverses both phenotypes, indicating a possible common basis. Finally, alternative σ factors may be implicated in stress resistance in *R. etli*. The *E. coli *extracytoplasmic stress factor σ^E^, implicated in the response to cell envelope stress, has recently been shown to be activated by ppGpp [25].

### Stationary phase behaviour

Besides in response to specific stresses, involvement of (p)ppGpp in stationary phase survival has been reported in many organisms. *rpoS*, whose expression and function is mediated by (p)ppGpp [26,27], is often important for both specific and general stress resistance. However, *R. etli *CNPAF512, like ε– and other α-proteobacteria, lacks this stress response factor. Currently, little is known about alternative factors that are involved in these bacteria. Nevertheless, we also observe decreased survival of the *rel*_*Ret*_ mutant in AMS succinate and in the early stationary phase in TY medium although cells still appeared intact. This response in TY medium was coupled with aberrant cell morphologies and a rise in the OD_600 _value, similar to *C. jejuni *[28]. However, upon a prolonged stationary phase, the colony forming ability of the wild type suddenly dropped in TY medium whereas it remained high in AMS succinate medium for the period studied. Based mainly on research in *E. coli*, three main models are proposed to account for this loss of reproduction during starvation. The VBNC (viable but non-culturable) – theory states that bacteria initiate a specific pathway generating dormant forms. The other theories suggest that cells become non-culturable due to cellular deterioration or through initiation of programmed cell death pathways, both finally leading to cell death [29]. With respect to cellular deterioration, increased protein oxidation during early stadia of starvation was identified as an important factor in *E. coli*. This oxidation process was tightly associated with the appearance of aberrant protein forms caused by an increased erroneous incorporation of amino acids [30]. In aerobic conditions, misfolded proteins are oxidized and are in turn responsible for elevated expression of the heat shock protein genes during starvation in *E. coli *[31]. As bacteria lacking (p)ppGpp fail to adapt their metabolism to starvation conditions, they accumulate misfolded and damaged proteins [3]. This could explain why viability is seriously impaired in the absence of (p)ppGpp as the cellular deterioration process will be enhanced. Accumulation of aberrant proteins might also be reflected in the aberrant cell morphologies observed in TY medium. Furthermore, in a *C. jejuni rel*_*Cje *_mutant nearly all heat shock genes were dramatically upregulated at the onset of the stationary phase when optical density also increases in the mutant [28]. Also, increased sensitivity towards oxidative stress as observed for *R. etli rel*_*Ret *_bacteroids and late stationary phase cultures might correlate with the increased presence of abnormal proteins, which are more susceptible to carbonylation. Another possibility is a relationship between (p)ppGpp and toxin-antitoxin (TA) modules as proposed by work on the *mazEF *and *relBE *operons in *E. coli*. Little information is available about the function of TA loci in rhizobia. Twelve possible TA loci have been identified on the *S. meliloti *chromosome [32], with genes homologous to *vapBC *located immediately downstream of the *rel*_*Sme *_gene. However, analysis of their function might be difficult as there is a high degree of redundancy (e.g. seven possible *vapBC *loci). In addition, from our sequencing results as well as the recently annotated genome sequence of the *R. etli *CFN42 chromosome, no evidence could be found for a TA locus downstream of the *rel*_*Ret *_gene.

### Cell morphology

From our results, it appears that cell morphology in *R. etli *is also regulated by intracellular levels of (p)ppGpp as the cell size of a *rel*_*Ret *_mutant is increased compared to the wild type once cultures enter the stationary phase as well as in cells differentiated to bacteroids. Similarly, ectopic production of (p)ppGpp in *Mycobacterium smegmatis *by overexpression of the *E. coli relA *gene results in a coccoid morphology of the bacteria in contrast to the normal bacilli form and was observed in low nutrient medium and late stationary phase cultures of *M. smegmatis *as well [33]. Colony and individual cell morphologies also differed between *M. smegmatis *and the *rel*_*Msm *_mutant, where significantly longer cells were observed with several of the elongated mutant cells containing multiple division septa in the cell [34]. In *Helicobacter pylori*, deletion of *spoT *results in premature transformation to a coccoid morphology [35]. Finally, in the ε-proteobacterium *C. jejuni*, mutation of *rel*_*Cje *_resulted in aberrant cell morphologies observed upon entry in the stationary phase with coccoid, significantly enlarged and electron dense cells. As observed for *R. etli*, this phenomenon was not reported for bacteria in the exponential phase, indicating that upon entry in the stationary phase, bacteria seem to display a defect in the process of cell division. In addition, the appearance of abnormal cell morphologies in *C. jejuni *also correlated with a sudden increase in OD_600 _values and CFU counts revealed a decrease in viability at the initial time points of the stationary phase compared to the wild type in agreement with our observations for *R. etli*. Although a role for (p)ppGpp in peptidoglycan biosynthesis and septum formation in *E. coli *was proposed in several studies [36-38], the molecular mechanisms involved are still unclear. In *E. coli*, it was found that inhibition of cell division by blocking or inactivating penicillin-binding protein 2, which regulates lateral elongation of peptidoglycan, can be relieved by increasing the intracellular concentration of ppGpp [39,40]. However, a direct effect of ppGpp at the level of transcription of *ftsZ*, involved in septum formation, was not found [41]. In *R. etli*, the target(s) of (p)ppGpp involved in the control of cell size are still unknown, however, reduction of the cell size may be an important adaptation of the strain for survival during starvation, in agreement with the observed reduction of cell size during starvation of *R. leguminosarum *[42].

### Symbiotic phenotype

TEM microscopic observation of sections of mature nodules of *rel*_*Ret *_mutant strains pointed to aberrant bacteroid morphology in both *R. etli *CNPAF512 and *R. etli *CE3 [10,11]. This was confirmed here for bacteroids isolated 5 weeks after inoculation. In addition, for all time points tested, bacteroid numbers obtained from plants inoculated with the mutant strain were consistently 10-fold lower compared to the wild type. This difference could not be clearly concluded from a previous TEM analysis [10]. Therefore, this difference might be attributed to the failure of a number of bacteroid cells to redifferentiate and form a colony, to a survival defect or to a lower number of bacteroids present in the nodule. Interestingly, [11] reported that for *R. etli *CE3 *rel*_*Ret*_, fewer plant cells in the nodule seemed to be invaded. However, a decreased resistance against oxidative stress, as observed here for *rel*_*Ret *_bacteroids, could also influence the number of CFU obtained at different time points during the symbiotic interaction. Oxidative stress, resulting from the presence of H_2_O_2_, hydroxyl and superoxide radicals, is an important stress factor in functioning nodules as ROS levels are high [15]. During senescence, the ROS concentration increases even further. For defence against ROS, organisms produce antioxidants and enzymes that prevent or repair oxidative damage. In *R. etli*, two genes involved in this defence have been described, *katG *and *prxS*, encoding a catalase-peroxidase and a peroxiredoxin respectively [43,14]. These genes partially overlap in function as mutation in both genes is necessary to affect nitrogen fixation [14]. However, *prxS*, located in an operon with *rpoN2*, is strongly expressed during the symbiotic interaction, mainly in an RpoN-dependent way. As decreased expression of σ^N^-dependent genes including the *rpoN2 *gene itself, is observed in *rel*_*Ret *_mutant bacteroids [10], this could indicate that (p)ppGpp contributes to symbiosis by redirecting gene expression in bacteroids favouring transcription of σ^N^-dependent genes. Besides an overall effect on the physiology, the strongly decreased nitrogen fixation ability and the decreased resistance against oxidative stress of the *R. etli rel*_*Ret *_mutant may result from a decreased expression of specific (symbiotic) target genes including σ^N^-dependent genes such as the *rpoN2 *gene, *prxS *and other nitrogen fixation genes. It will be important in future experiments to address the question whether (p)ppGpp may redirect gene expression in bacteroids favouring transcription of σ^N^-dependent genes. Indeed, it was recently shown using an *in silico *approach for the detection of -24/-12 type of promoters in Rhizobiales that the σ^N ^regulon may control more genes than traditionally assumed [44]. Interestingly, *S. meliloti *class I suppressors carry mutations in a region previously implicated in σ factor recognition [9].

Upon examination of stress resistance, bacteroids, isolated either at 35 or 42 days post inoculation, differ considerably from free-living bacteria. In general, in the presence of temperature, salt or oxidative stress, wild type bacteroids display a higher resistance compared to exponentially growing or stationary phase cells. Also, the response of the *rel*_*Ret *_mutant against these stresses differs. Overall, while exponentially growing or stationary phase *rel*_*Ret*_^- ^cells display increased sensitivity to heat, salt or H_2_O_2 _compared to the wild type, this is not the case in bacteroids. These results indicate that bacteroid physiology is profoundly different from free-living cells.

## Conclusion

Here, the central role of the previously identified *rel*_*Ret*_ gene region in cellular metabolism and stress resistance was further investigated. We demonstrate stress-dependent phenotypic differences between the wild type and *rel*_*Ret *_mutant. Furthermore our observations point to clear differences between the physiological status of free-living and symbiotic bacteria in relation to stress resistance. Taken together, our data demonstrate that (p)ppGpp-mediated regulation is important for physiological adaptation of free-living bacteria subjected to metabolic or stationary phase stresses and of *R. etli *bacteroids to the conditions prevailing in the nodules.

## Methods

### Bacterial strains and culture conditions

The bacterial strains and plasmids used in this work are listed in Table 2. *R. etli *strains were cultured in complex TY medium (0.3% yeast extract, 0.5% tryptone, 7 mM CaCl_2_) or minimal AMS medium at 30°C [45]. AMS medium was supplemented with carbon and nitrogen sources at a concentration of 10 mM, unless otherwise indicated. Antibiotics were supplied at the following concentrations: spectinomycin 50 μg ml^-1^, nalidixic acid 30 μg ml^-1^, neomycin 60 μg ml^-1 ^or tetracycline 1 μg ml^-1^.

**Table 2:** Bacterial strains and plasmids

**Strain or Plasmid**	**Description^a^**	**Source or reference**
**Strains**		

*Rhizobium etli*		

CNPAF512	Nal^r^, wild-type	Michiels *et al*., 1998a

CMPG8705	Sp^r^, *rel*_*Ret*_:: Ω-Sp, opposite orientation	This work

**Plasmids**		

pFAJ1702	Ap^r ^Tc^r^, stable RK2-derived cloning vector	Dombrecht *et al*., 2001

pCMPG8715	*rel*_*Ret *_gene in pFAJ1702	This work

^a ^Nal^r^: nalidixic acid resistance; Ap^r^: ampicillin resistance; Tc^r^: tetracycline resistance

### Plant experiment and bacteroid isolation

Seeds of *Phaseolus vulgaris *cv. Limburgse vroege were sterilized and germinated as previously described [46]. Plants were inoculated with 100 μl of an overnight bacterial culture resuspended at an OD_600 _of 0.4 in 10 mM MgSO_4 _[47]. For each *R. etli *strain at least 10 plants were inoculated. Snoeck medium was used to grow the common bean plants [48]. Plants were grown in a plant growth room with a 12 h photoperiod (day/night temperature 26°C/22°C; relative humidity 65–70%). Nitrogenase activity was determined by measuring acetylene reduction activity of nodulated roots in a closed vessel three weeks after inoculation. Samples were analyzed by a Hewlett-Packard 5890A gas chromatograph equipped with a 'PLOT fused silica' column and an HP3396A integrator. Propane was used as an internal standard. For stress experiments, bacteroids were purified from the nodules by differential centrifugation [49] and subsequently resuspended in 10 mM MgSO_4_.

### Growth experiments and bacterial survival during stress

To study bacterial growth over an extended period of time, overnight cultures of the strains were washed and diluted to an optical density (at 600 nm) of 0.5 in 10 mM MgSO_4_. Subsequently, these cultures were diluted 100-fold in 10 mM MgSO_4 _after which 295 μl of the growth medium (TY or AMS medium supplemented with carbon and nitrogen sources at 10 mM concentrations) was inoculated with 5 μl of the suspension (dilution approximately 6000-fold). Optionally, medium was supplemented with NaCl as indicated in the text. To study temperature sensitivity, growth was monitored at 37°C. The optical density was measured automatically at 600 nm every 30 min in a Bioscreen C (Labsystems Oy) during at least 4 days. For each time point, the average optical density was calculated from 5 independent measurements. Experiments were repeated at least twice.

To study stress survival, an overnight culture was used to inoculate 5 ml TY cultures with a dilution factor ranging from 1/10 to 1/10,000. After overnight incubation, the optical density of these TY cultures at 600 nm was determined and for a range of optical densities between 0.1 and 1, appropriate cultures were selected. Subsequently, a volume of 0.5 ml of the selected samples was placed in a 30°C water bath for one hour in the presence of 10 mM H_2_O_2_, in a 30°C incubator (shaking at 200 rpm) for 1.5 h and 6 h in the presence of 2.5 M NaCl, or in a water bath at 45°C. Samples were removed at the indicated time points, dilution series were prepared in 10 mM MgSO_4 _and plated on TY plates containing nalidixic acid. Control samples were incubated without the stress agent at 30°C and the control CFU numbers were determined at the 0 h timepoint as well as at the same timepoint as the stressed samples. Colonies were scored after three days incubation at 30°C. The total number of colony forming units (CFU) per ml culture was calculated. Experiments were repeated at least three times.

To study survival, wild-type and mutant strains were precultured in TY medium. Subsequently, cell pellets were washed and resuspended in the indicated medium at an optical density at 600 nm of 0.4. A volume of 100 ml culture medium was then inoculated with 1 ml of this suspension and incubated at 30°C. Samples of 1 ml were removed at the indicated time points and 10-fold dilution series were prepared and plated on TY or AMS succinate plates or on the same plates containing 30 μg ml^-1 ^nalidixic acid. Colonies were scored after three days incubation at 30°C. For a number of time points, heat (45°C) sensitivity, oxidative stress (10 mM H_2_O_2_) and NaCl sensitivity (2.5 M) were tested as described above.

For bacteroid experiments, three samples were tested for each strain. To prepare a sample, nodules from 3 different inoculated plants were isolated, weight was determined and bacteroids were isolated [49] and suspended in 4.5 ml 10 mM MgSO_4 _(approximately equal numbers). Stress survival of bacteroids in the presence of 10 mM H_2_O_2 _or 2.5 M NaCl or at 45°C was determined as described for free-living bacteria. Finally, bacteroids were diluted in MgSO_4 _before plating. Values were calculated as CFU per gram nodule.

### Microscopy

Light microscopic examination of bacteria was done on a Nikon Optiphot-2 microscope equipped with a fluorescence unit after staining of the bacteria (diluted in 10 mM MgSO_4_), with the LIVE/DEAD BacLight Bacterial Viability kit (Molecular Probes). 1 μl of each staining solution (SYTO 9 and propidium iodide) was added to 600 μl of the cell suspension and incubated for 15 min in the dark. Samples were placed on poly-lysine coated glass plates or concentrated by filtration on a 0.1 μm filter (Millipore). Images were taken using a digital DS camera head DS-5M (Nikon) controlled by a DS Camera Control Unit DS-L1.

For transmission electron microscopy (TEM) analysis, bacterial cells were adsorbed to uncoated grids. The grids were placed on a drop of bacterial suspension for 15 seconds, then incubated in 0.25% phosphotungstenic acid (pH 7) for 30 seconds, washed several times and excess liquid was drained. The bacteria were observed with a Philips EM 208S transmission electron microscope at 80 kV. Images were digitized using the SISR image analysis system.

## Authors' contributions

KB, SB and MF performed the experiments. KB and JM conceived the study and contributed to the interpretation of the data. IL performed the TEM analysis. KB, JM, MF and MV were involved in drafting the manuscript. All authors read and approved the final manuscript.
